# A review on the reporting and assessment of adverse effects associated with high-definition transcranial direct current stimulation

**DOI:** 10.3389/fnhum.2025.1682771

**Published:** 2025-12-05

**Authors:** Tiffany Carther-Krone, Ji Hyun Ko

**Affiliations:** 1 Department of Human Anatomy and Cell Science, Max Rady College of Medicine, University of Manitoba, Winnipeg, MB, Canada; 2 PrairieNeuro Research Centre, Kleysen Institute for Advanced Medicine, Winnipeg Health Science Centre, Winnipeg, MB, Canada; 3 Graduate Program in Biomedical Engineering, Price Faculty of Engineering, University of Manitoba, Winnipeg, MB, Canada

**Keywords:** HD-tDCS, safety, tolerability, adverse event, brain stimulation

## Abstract

**Background:**

High-definition transcranial direct current stimulation (HD-tDCS) is a non-invasive brain stimulation technique that offers increased spatial precision compared to conventional tDCS. As its use has expanded across research and clinical settings, there has been increasing interest in understanding its safety and tolerability.

**Objective:**

This review summarizes adverse events related to HD-tDCS in both healthy and clinical populations, focusing on how stimulation intensity, session frequency, and polarity influence tolerability.

**Results:**

In healthy populations, HD-tDCS is most often administered at 1–2 mA for 20 min. The most reported adverse events include tingling, itching and burning localized to the site of stimulation, typically described as mild or transient. Studies comparing active and sham stimulation generally report no significant differences in adverse event frequency or intensity, even at higher intensities of 2–3 mA. Reports of severe adverse events are rare, and participant dropout due to discomfort is uncommon. Multi-session protocols show similar safety profiles, suggesting that repeated stimulation does not increase adverse effects. In clinical populations HD-tDCS is typically delivered across multiple sessions. Reported adverse events are mild and transient, with few reports of severe outcomes. Polarity-specific comparisons suggest that anodal and cathodal stimulation are similarly tolerated, with no notable differences in adverse event profiles.

**Conclusion:**

Overall, current evidence indicates that HD-tDCS is a safe and well-tolerated technique across diverse populations and stimulation parameters. Continued use of standardized adverse event reporting will be important to further confirm these findings as clinical application broaden.

## Highlights

- Healthy and clinical populations were not found to be particularly vulnerable to HD-tDCS.
- The most commonly reported adverse effects were itching, tingling and burning sensation, typically described as mild or transient.
- Common adverse effects were localized to the site of stimulation.
- Multi-session protocols show similar safety profiles to single-session protocols.

## Background

**Figure 1:**
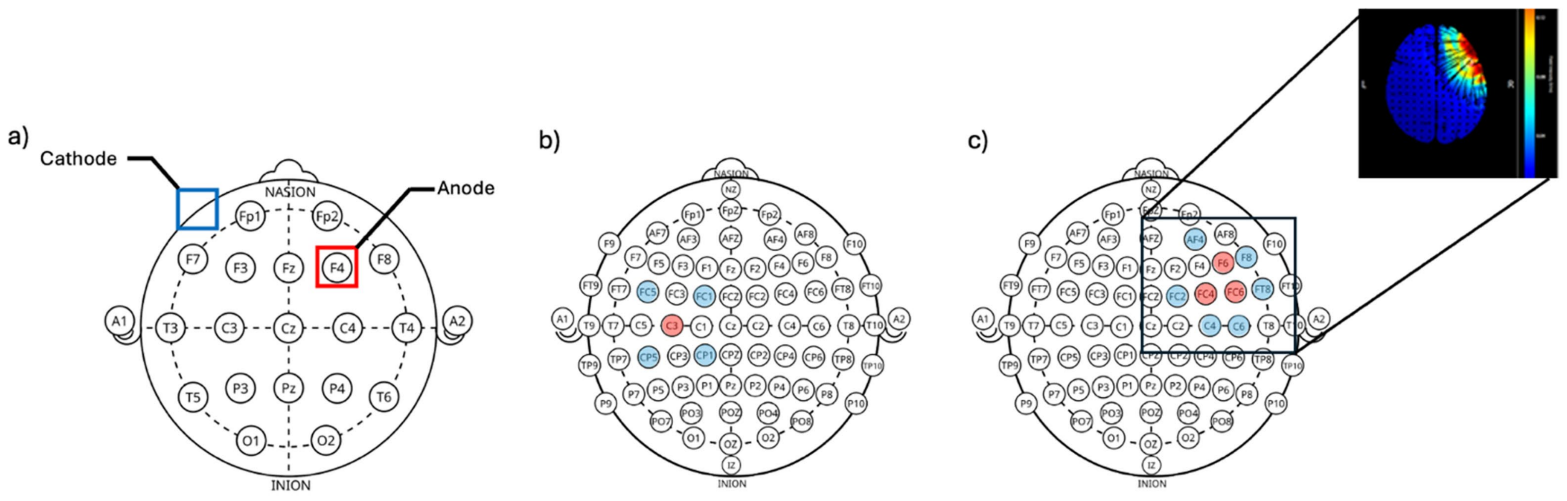
Comparisons of electrode configurations in transcranial direct current stimulation (tDCS). **(a)** In conventional tDCS, two large rubber electrodes are placed in saline-soaked sponges over the scalp. Current flows from the anodal electrode placed over the target brain region to a return electrode positioned over another brain region or an extracranial area. **(b)** High-definition tDCS (HD-tDCS) increases focality by using smaller electrodes arranged in specific configurations. One commonly used setup is the 4×1 ring montage, in which a central electrode is surrounded by four return electrodes arranged in a circular pattern to constrain current flow and improve focality. **(c)** More complex HD-tDCS configurations use montages with more than five electrodes, involving multiple anodal and cathodal sites. Here the right dorsolateral prefrontal cortex is targeted. The cutout shows the simulated electrical current in a head model based on magnetic resonance imaging using HDTargets (Soterix, Inc).

Over the last 20 years non-invasive brain stimulation (NIBS) techniques, such as transcranial direct current stimulation (tDCS), have received increased interest (Ruffini et al., 2013). In conventional tDCS two rubber electrodes are placed in saline-soaked sponges over the scalp such that the ingoing current projects from an electrode placed over the target brain region to a return electrode placed over a different brain region or an extracranial area (Figure 1a). This setup delivers a relatively weak current through the cortex, aiming to modulate brain function (Pascual-Leone and Fregni, 2007; Nitsche et al., 2007). However, the size of the tDCS electrodes/sponges has resulted in relatively non-focal stimulation (Lang et al., 2005), and often affects not only the targeted region but also intervening areas between the two electrodes, as demonstrated by computational modeling studies (Datta et al., 2009; Faria et al., 2011).

High-definition tDCS (HD-tDCS) is a relatively recent modification of conventional tDCS that increases focality by using multiple smaller electrodes (Kuo et al., 2013; Villamar et al., 2013a). While electrode configurations are often guided by computational modeling approaches to optimize current delivery to the target region (Edwards et al., 2013; Dmochowski et al., 2011), one of the most commonly used montage set-ups is the “4×1 ring montage,” in which a central electrode is surrounded by four return electrodes arranged in a circular formation (Figure 1b). The configuration serves to constrain current flow to the region between the center and ring electrodes (Villamar et al., 2013a) and improve the focality of stimulation compared to the traditional bipolar montage of conventional tDCS (Datta et al., 2009; Edwards et al., 2013). Another commonly used configuration involves montages with more than five electrodes, in which multiple anodal and cathodal sites are defined to steer and confine current flow toward targeted brain regions (Figure 1c). These more complex arrangements further enhance focality and allow for greater precision in guiding the current flow.

This increased focality of HD-tDCS, along with its non-invasiveness and its ability to induce changes in cortical excitability that may outlast those of conventional tDCS (Kuo et al., 2013) has contributed to its growing popularity in both cognitive neuroscience and clinical research. HD-tDCS has been most commonly applied to modulate a variety of cognitive functions including attention (Lu et al., 2020; Nikolin et al., 2019; Martínez-Pérez et al., 2020), memory (Nikolin et al., 2019; Nikolin et al., 2015; Chua et al., 2017), and executive functions (Imburgio and Orr, 2018; Lu et al., 2021). Clinically, it has been investigated in neuropsychiatric and neurological conditions such as fibromyalgia (Villamar et al., 2013b; Castillo-Saavedra et al., 2016), mild cognitive impairment (Rezakhani et al., 2024; Iordan et al., 2022), post-traumatic stress disorder (Hampstead et al., 2020), depression (Salehinejad et al., 2023; Wong et al., 2019), and tinnitus (Jacquemin et al., 2021). The use of the 4×1 ring montage is particularly common in these contexts because it provides improved focality over conventional bipolar tDCS, allowing researchers to target relatively constrained cortical regions such as the dorsolateral prefrontal cortex. More complex multi-electrode montages have been motivated by the need to direct current toward distributed but functionally connected cortical regions, thus expanding the potential applications of HD-tDCS. Direct comparisons of HD-tDCS and conventional tDCS remain relatively limited but generally support comparable safety and tolerability. For example, Jacquemin et al. reported that fewer sensations were experienced during HD-tDCS than conventional tDCS in 39 patients with tinnitus (Jacquemin et al., 2018), while da Silva Machado et al. found that both techniques were similarly well tolerated in 12 high endurance athletes (da Silva Machado et al., 2021). To date, a few studies have demonstrated superior or faster benefits of HD-tDCS over conventional tDCS in clinical populations (Zeng et al., 2024; Jog et al., 2025), but more studies need to be done for a proper meta-analysis.

Despite the increasing use of HD-tDCS, its safety profile remains less established than that of conventional tDCS. While the majority of reported adverse effects in conventional tDCS are mild and transient, such as tingling or itching under the electrodes, some studies have noted more persistent effects—most commonly skin irritation—that may last beyond the stimulation period. Given differences in electrode configurations, current density distributions, and overall stimulation profiles, safety findings from conventional tDCS cannot be assumed to directly translate to HD-tDCS.

The aim of this review is to summarize the current literature on adverse events associated with HD-tDCS in both healthy and clinical populations, focusing on the impact of stimulation parameters, number of HD-tDCS sessions and polarity. By consolidating available evidence, this review seeks to provide researchers and clinicians with clearer insight into the tolerability and risks associated with HD-tDCS, and to support the ongoing development of evidence-based safety guidelines for its use.

## Adverse effects of HD-tDCS

Since the introduction of HD-tDCS in 2009, safety and tolerability have been at the forefront of study design protocols. Early work focused on optimizing “high-definition” electrode-gel parameters for electrode durability, skin safety, and subjective pain, testing various parameters such as anode and cathode electrode potential, temperature, pH, and subjective sensation during the application of 2 mA direct current, for up to 22 min, on each subjects’ forearms (Minhas et al., 2010). Using this protocol, no serious adverse events such as skin burning were observed. Minor adverse effects included transient redness at the site of the electrodes, skin irritation in the form of small bumps of black dots (<1 mm) and apparent roughening of the skin under the electrodes. All effects on the skin were reversible and disappeared within a few hours, and no subject reported lasting irritation or pain.

Following computational modeling studies suggested that replacing the two large sponge electrodes used in conventional tDCS with an array of smaller electrodes could improve targeting (Dmochowski et al., 2011). Subsequent research using a 4 × 1 ring configuration demonstrated that this targeted montage could successfully induce changes in neuronal excitability while maintaining a favorable safety profile (Caparelli-Daquer et al., 2012). Participants reported only mild sensations like itching or tingling, and none requested to terminate stimulation. Building on these early demonstrations of safety and tolerability, a growing number of studies have since applied HD-tDCS across diverse healthy and clinical populations, consistently reporting minimal and transient side effects.

### Healthy populations

HD-tDCS is generally well tolerated in healthy adults, with adverse events that are mild, transient, and comparable to conventional tDCS. The most frequently reported sensations include tingling, itching, or a mild burning sensation at the site of the electrode (Table 1). These sensations typically occur in both active and sham conditions and are well tolerated. Across studies, serious adverse events are rare, and discomfort typically does not interfere with task performance or study completion. Nonetheless, differences in stimulation parameters, session number, and polarity may influence the occurrence of side effects, and a thorough examination of these parameters is critical for guiding safe research and clinical applications.

**Table 1:** Study design and adverse event reports for healthy populations.

	Sample size and population	Stimulation parameters	Number of sessions	Polarity	Reported adverse events
Caparelli-Daquer et al. (2012)	10 healthy adults	1 mA, 20 min 4×1 ring montage with anode centered at the hand area of the motor cortex	3 sessions	Both	Transient sensation (itching/tingling) under the electrodes
			1 anodal		
			1 cathodal		
			1 sham		
Borckardt et al. (2012)	24 healthy adults	2 mA, 20 min motor cortex	1 session	Anodal	Itchiness, tingling, prickling, pressure, stinging, uncomfortable, warm
	Active (*n* = 13)				
	Sham (*n* = 11)				
Kuo et al. (2013)	14 healthy adults	2 mA, 10 min 4x1 montage with center at right ADM muscle	2 sessions	Both	Tingling sensation
			1 anodal		
			1 cathodal		
Roy et al. (2014)	8 healthy adults	1 mA, 10 min 4x1 montage with center at left sensorimotor cortex	3 sessions	Both	Burning/tingling sensation under region of electrodes
			1 anodal		
			1 cathodal		
			1 sham		
Brunyé et al. (2014)	24 healthy adults	0.5 / 2 mA, 15 min 4x1 ring montage with anode centered at the left DLPFC (FC3)	4 sessions	Anodal	Perceived sensation ratings higher in active 2 mA vs.1.5 mA condition at 30s after stimulation start, but converged over time
			2 active (0.5 mA / 2 mA)		
			2 sham (0.5 mA / 2 mA)		
Nikolin et al. (2015)	16 healthy adults	2 mA, 20 min 4x1 montage with center at left DLPFC (F3) / planum temporale (Cp5); Montage at left medial temporal lobe	4 sessions	Anodal	Erythema, mild stinging, itching, irritation; Two self-resolving headaches (one from sham); One participant withdrew due to “needle prickling” sensation
			3 active		
			1 sham		
Garnett and den Ouden (2015)	31 healthy adults	2 mA, 20 min Left posterior superior temporal gyrus	3 sessions	Both	No difference between active/sham groups in ratings of pain/unpleasantness
			1 anodal		
			1 cathodal		
			1 sham		
Price et al. (2016)	18 healthy adults	2 mA, 20 min 4x1 montage with anode centered at either the left angular gyrus, AG (CP5) or right AG (CP6)	3 sessions	Anodal	No differences between stimulation conditions in ratings of tingling (M = 2.45/10) or burning (M = 2.14/10) sensations
			2 active (left/right AG)		
			1 sham		
Flood et al. (2016)	30 healthy males	2 mA, 10 min 4x1 montage with anode centered over the left motor cortex (C3)	2 sessions	Anodal	Procedure well tolerated with no reports of negative side effects from participants
			1 active		
			1 sham		
Gbadeyan et al. (2016b)	30 healthy adults	1 mA, 20 min 4x1 montage with center at right DLPFC (F4)	2 sessions	Anodal	No difference between active/sham groups in ratings of headache, neck/scalp pain, tingling, itching, burning, sleepiness, concentration, mood changes, skin irritation
			1 active		
			1 sham		
Gbadeyan et al. (2016a)	120 healthy adults	1 mA, 20 min concentric setup with anode centered at the left or right DLPFC (F3/F4) or left or right M1 (C3/C4)	2 sessions	Anodal	No difference between active/sham groups in ratings of headache, neck/scalp pain, tingling, itching, burning, sleepiness, concentration, or mood changes; All participants tolerated stimulation well
	Left DLPFC (*n* = 30)		1 active		
	Right DLPFC (*n* = 30)		1 sham		
	Left M1 (*n* = 30)				
	Right M1 (*n* = 30)				
Shen et al. (2016)	39 healthy adults	2 mA, 20 min 4x1 montage with electrode centered at either the left DLPFC (F3) or right DLPFC (F4)	3 sessions	Both	All participants tolerated the stimulation well
	Exp. 1 (*n* = 18)		Exp.1: L/R anodal/sham		
	Exp. 2 (*n* = 21)		Exp.2: L/R cathodal/sham		
Turski et al. (2017)	5 healthy adults	1/1.5 mA, 20 min varied networks targeted	20 sessions	Anodal	Tingling, transient redness at stimulation site, perception of continued stimulation following session; one self-resolving headache
Chua et al. (2017)	39 healthy adults	2 mA, 20 min 4x1 ring montage with anode centered at the left DLPFC (F3) or left anterior temporal lobe (T7)	3 sessions	Anodal	DLPFC Stimulation: 1 severe headache, 4 severe tingling, 6 severe burning, 1 severe redness ATL Stimulation: 2 severe tingling, 1 severe burning, 1 severe sleepiness; Sham: 2 severe burning 1 participant withdrew after “pre-stim tickle”
	Left DLPFC (*n* = 13)				
	Left ATL (*n* = 13)				
	Sham (*n* = 13)				
Pixa et al. (2017a, 2017b)	32 healthy adults	1 mA, 15 min montage targeted to both primary motor cortices (M1/M2)	1 session	Anodal	No difference between active/sham groups in ratings of fatigue or discomfort
	Active (*n* = 16)				
	Sham (*n* = 16)				
Hill et al. (2017)	19 healthy adults	1 mA, 20 min 4x1 montage with center at left DLPFC (F3)	3 sessions	Anodal	Higher rating of itchiness for HD- compared to BP-tDCS and sham groups after 5 min of stimulation; no difference in ratings of burning or tingling at 5 min; no differences in ratings of itchiness, burning or tingling after 15 min
			2 active (BP/HD-tDCS)		
			1 sham		
Lenoir et al. (2017)	28 healthy adults	1 mA, 20 min	1 session	Cathodal	Moderate tingling/itching sensation at the site of the electrodes
	Active (*n* = 14)	4x1 ring montage with cathode centered at the			
	Sham (*n* = 14)	left (C3) or right (C4) sensorimotor cortex			
Martin et al. (2017a, 2017b)	40 healthy adults	1 mA, 20 min 4x1 Ring configuration with center at dorsomedial PFC	2 sessions	Anodal	No difference between active/sham groups in ratings of headache, neck pain, tingling, itching, burning, sleepiness, concentration, mood changes; Anodal stimulation rating scalp pain higher than sham group
*2 experiments			1 active		
			1 sham		
Perceval et al. (2017)	50 healthy adults	1 mA, 20 min 4x1 ring montage with anode centered over the left posterior temporal lobe (CP5)		Anodal	No difference between active/sham groups in ratings of headache, neck pain, tingling, itching, burning, redness, sleepiness, concentration or mood change
	Active (*n* = 25)				
	Sham (*n* = 25)				
Guo et al. (2018)	58 healthy adults	1.5 mA, 20 min 4x1 montage with center at left DLPFC (F3)	1 session	Both	Burning/itching sensation; No difference in pain ratings between groups; no side effects reported a day after the end of the experiment
	Anodal (*n* = 20)				
	Cathodal (*n* = 16)				
	Sham (*n* = 22)				
To et al. (2018)	45 healthy adults	1 mA, 20 min 5 electrode montage targeting the dACC (Fz)	1 session	Both	Tingling (active: 33.33% / sham: 58.33%)
	Anodal (*n* = 15)				Itching (active: 33.33% / sham: 33.33%)
	Cathodal (*n* = 15)				Burning (active: 22.22% / sham: 13.33%)
	Sham (*n* = 15)				HD-tDCS well tolerated; no complications noted
Reckow et al. (2018)	101 older adults	2 mA, 20–30 min (*n* = 66, 31 active)	Varied multi-sessions	Both	Higher sleepiness ratings in active compared to sham groups; no difference between groups in ratings of burning, tingling, itching
		3 mA, 20–30 min (*n* = 35, 20 active)			
		Varied montages			
Hill et al. (2018)	16 healthy adults	1.5 mA, 15 min Montage targeted left DLPFC or left DLPFC + parietal cortex	3 sessions	Anodal	No difference between groups in ratings of cutaneous sensations; one participant withdrew after first session due to discomfort during stimulation
			2 active		
			1 sham		
Naka et al. (2018)	20 healthy adults	1.5 mA, 16 min 4x1 montage with center at left DLPFC (F3)	1 session	Anodal	No difference between active/sham groups in ratings of headache, neck/scalp pain, tingling, itching, burning, sleepiness, concentration, mood changes
	Active (*n* = 10)				
	Sham (*n* = 10)				
Hill et al. (2019)	20 healthy adults	1.5 mA, 15 min 4x1 ring montage with the anode centered at the left DLPFC (F3)	3 sessions	Anodal	No difference in ratings of cutaneous sensations between stimulation groups
			Active (2 sessions)		
			Sham (1 session)		
Besson et al. (2019)	15 healthy adults	2 mA, 20 min 4x1 montage with the anode centered at the left sensory motor cortex (C3)	3 sessions	Anodal	No difference in ratings of cutaneous sensations between stimulation groups
			Active (2 sessions)		
			Sham (1 session)		
Weintraub-Brevda and Chua (2019)	60 college students	2 mA, 20 min 4x1 montage with anode centered at the left VLPFC (F7) or the right VLPFC (F8)	1 session	Anodal	Right VLPFC: 4 severe tingling, 2 severe sleepiness, 1 severe burning
	Left VLPFC (*n* = 20)				Sham: 2 severe burning; No difference between groups in ratings of headache, neck/scalp pain, tingling, itching, burning, sleepiness, concentration, mood changes
	Right VLPFC (*n* = 20)				
	Sham (*n* = 20)				
Lang et al. (2019)	60 healthy adults	2 mA, 10 min 4x1 montage with anode centered on the right fusiform cortex (P10)	1 session	Anodal	No difference between stimulation groups in ratins of itching, burning or discomfort; Ratings of tingling sensations were higher in the HD-tACS group compared to HD-tDCS and sham groups
	HD-tACS (*n* = 20)				
	HD-tDCS (*n* = 20)				
	Sham (*n* = 20)				
Martin et al. (2019a, 2019b)	52 healthy adults	1 mA, 20 min ring configuration with center at dorsomedial PFC or rTPJ	2 sessions	Anodal	No evidence for effect of stimulation on adverse effects
**2 experiments			1 active		
			1 sham		
Savic et al. (2019)	89 healthy adults	2 mA, 20 min 4x1 montage with center at left DLPFC (F3) or right DLPFC (F4)	1 session	Both	Higher rating of pain for active (1.7/10) compared to sham (0.7/10) group; no difference between groups in ratings of unpleasantness
	Anodal L. DLPFC (*n* = 16)				
	Anodal R. DLPFC (*n* = 15)				
	Cathodal L. DLPFC (*n* = 16)				
	Cathodal R. DLPFC (*n* = 11)				
	Sham L. DLPFC (*n* = 16)				
	Sham R. DLPFC (*n* = 15)				
Choi and Perrachione (2019)	60 healthy adults	2 mA, 13 min 6 electrode montage with center targeted at the left superior temporal cortex (T7 and TP7)	1 session	Both	Reports of scalp sensations did not differ between sham and active groups; Reported of mild to moderate tingling, mild pain, and mild burning sensations
	Anodal (*n* = 20)				
	Cathodal (*n* = 20)				
	Sham (*n* = 20)				
Wen et al. (2019)	Exp.1: 56 healthy adults	1.5 mA, 20 min 4x1 montage with cathode centered at left DLPFC (F3)	1 session	Cathodal	All participants reported no or very tolerable symptoms
	Exp.2: 60 healthy adults				
	Active (*n* = 30)				
	Sham (*n* = 30)				
Gbadeyan et al. (2016a, 2016b)	36 healthy adults	1 mA, 20 min ring configuration with center at right DLPFC (F4)	2 sessions	Anodal	No difference between active/sham groups in ratings of headache, neck pain, tingling, itching, burning, sleepiness, concentration, mood changes; Stimulation tolerated well
			1 active		
			1 sham		
Gan et al. (2019)	22 healthy adults	2 mA, 20 min 4x1 montage with center at left PPC (P3)	4 sessions	Anodal	One participant reported severe tingling during stimulation; All others were mild/moderate reports of tingling, itching, burning, pain, headache; 16 participants reported mild tingling, itching, burning, pain, headache during sham; One participant reported moderate headache following sham
			HD-tDCS		
			iTBS		
			ProcTBS		
			Sham		
Donaldson et al. (2019)	53 healthy adults	2 mA, 20 min 4x1 montage with central electrode centered at the right temporoparietal junction (P6)	1 session	Both	Two participants temporarily stopped stimulation due to discomfort, but completed the stimulation after a short break; Mild–severe ratings of itching, tingling, burning, headache, neck/scalp pain, numbness, fatigue, thinking difficulties
	Anodal (*n* = 17)				
	Cathodal (*n* = 18)				
	Sham (*n* = 18)				
Zhou et al. (2019)	47 women	2 mA, 20 min 4x1 montage with the anode centered at the left DLPFC (F3)	2 sessions	Anodal	Discomfort, itching, tingling during initial stimulation period
	23 with high-avoidant attachment		Active		
	24 with low-avoidant attachment		Sham		
Nikolin et al. (2019)	78 healthy adults	2 mA, 20 min 4x1 montage with center at left IPS (P3) or left DLPFC (F3)	1 session	Anodal	Mild tingling, itching, burning, stinging; Higher rating of pain for DLPFC group compared to IPS and Sham groups; No differences in ratings of tingling, itching, burning; Two participants withdrew due to stinging/burning sensations
	IPS Active (*n* = 26)				
	DLPFC Active (*n* = 26)				
	Sham (*n* = 26)				
Ke et al. (2019)	30 healthy adults	1.5 mA, 25 min 4x1 montage with center at left DLPFC (F3)	5 sessions	Anodal	Slight tingling sensation under the electrode
	Active (*n* = 15)				
	Sham (*n* = 15)				
Hartmann et al. (2020)	18 healthy adults	2 mA, 25 min 4x1 montage with anode centered at the left (P3) or right (P4) intraparietal sulcus	3 sessions	Anodal	No difference in ratings of comfort/discomfort or pain across stimulation conditions; Pain ratings generally low
			2 Active		
			1 sham		
Behroozmand et al. (2020)	Exp. 1: 30 healthy subjects	2 mA, 20 min 5 electrodes configured to induce focal stimulation of the left ventral motor cortex	1 session	Both	No difference in ratings of pain or discomfort across stimulation groups or across time; Sensations gradually reduced across time; no subjects reported feeling any unusuall discomfort or requested that the stimulation be stopped
	Anodal (*n* = 10)				
	Cathodal (*n* = 10)				
	Sham (*n* = 10)				
	Exp. 2: 26 healthy subjects	1 mA or 2 mA, 20 min 5 electrodes configured to induce focal stimulation of the left ventral motor cortex	1 session		
	1 mA Active: (*n* = 13)				
	2 mA Active: (*n* = 13)				
Kumar et al. (2020)	96 healthy participants	2 mA, 15 min 4x1 ring configuration with the cathode centered at the left (P3) or right (P4) PPC		Cathodal	Participants reported no discomfort with the HD-tDCS
Sedgmond et al. (2020)	55 healthy participants	1.5 mA, 20 min 4x1 ring configuration with the anode over the right DLPFC (F4)	2 sessions	Anodal	Higher ratings of pain/discomfort in active compared to sham stimulation groups; average ratings in both were low Dizziness, headache and skin irritation noted in follow-up questionnaire
			Active		
			Sham		
Xiao et al. (2020)	14 healthy adults	2 mA, 20 min 4x1 montage with center at Cz	2 sessions	Anodal	No side effects or risk events were reported by participants
			1 active		
			1 sham		
Splittgerber et al. (2020)	24 healthy adults	1 mA, 20 min 5 electrode montage targeting the left DLPFC	3 sessions	Anodal	No differences between conditions in incidence or intensity of itching, pain, burning, warmth/heat, metallic/iron taste, fatigue/decreased alertness
			2 active (BP/HD-tDCS)		
			1 sham		
Ballard et al. (2021)	49 healthy adults	2 mA, 20 min Montage of 8 electrodes targeting the left DLPFC (F3)	2 sessions	Both	Mild ratings across groups for itching, pain, burning, warmth/heat pinching, metallic/iron taste, fatigue; 3 participants withdrew due to discomfort
	Cathodal/Sham (*n* = 25)		1 active		
	Anodal/Sham (*n* = 24)		1 sham		
Lerner et al. (2021)	60 healthy adults	1.5 or 2 mA, 20 min Montage of 9 electrodes targeting the primary motor cortex	1 session	Anodal	Tingling, burning, itching, hair pulling, irritation frequency of adverse effects did not differ between groups, but strength of discomfort was higher for stimulation vs. sham (no difference between 1.5/2 mA groups)
	2 mA group				
	1.5 mA group				
	Sham group				
Huang et al. (2021)	84	1 mA, 10 min; two electrode montages: (1) targeting aDMN using FpZ as stimulation electrode and (2) pDMN using Pz as stimulation electrode	1 session	Both	Mild ratings across groups for headache, neck/scalp pain, tingling, itching, burning, sleepiness, concentration, mood changes, skin irritation; no stimulation complications noted by participants or experimenters
	aDMN (*n* = 40)				
	Anodal/Cathodal/Sham				
	pDMN (*n* = 44)				
	Anodal/Cathodal/Sham				
da Silva Machado et al. (2021)	12 high endurance athletes	2.4 mA, 20 min 9 electrode montage set to increase focality to the motor cortex	3 sessions	Anodal	No differences in ratings of itching, pain, burning, pinching, warmth/heat, metallic/iron taste or fatigue between stimulation groups; tDCS was well-tolerated
			Conventional tDCS		
			HD-tDCS		
			Sham		
Wan et al. (2021)	35 healthy adults	2 mA, 20 min 4x1 montage with center at left primary motor cortex (M1)	2 sessions	Anodal	No side effects or risk events were reported by participants
			1 active		
			1 sham		
Dai et al. (2021)	24 healthy adults	1.5 mA, 20 min 4x1 montage with cathode centered at left M1	2 sessions	Cathodal	Procedure was well tolerated, no adverse effects experienced during or after intervention
			1 active		
			1 sham		
Martin et al. (2021)	52 healthy adults	1 mA, 20 min Ring configuration with center at dorsomedial PFC or rTPJ	2 sessions	Anodal	No difference in total adverse effects between active/sham groups
			1 active		
			1 sham		
Kold and Graven-Nielsen (2021)	81 healthy adults	2 mA, 20 min Montage of 4 electrodes targeting the left DLPFC (F3); Montage of 5 electrodes targeting M1 (C3); Montage of 7 electrodes targeting both left DLPFC and M1 (F3/C3)	3 sessions	Anodal	No adverse effects of the interventions were reported by participants
	DLPFC (*n* = 21)				
	M1 (*n* = 20)				
	DLPFC & M1 (*n* = 20)				
	Sham (*n* = 20)				
Lu et al. (2021)	43 undergraduate students	1.5 mA, 20 min 4x1 montage with anode centered at the left DLPFC (F3)	9 sessions	Anodal	Slight skin tingling
	Active (*n* = 22)				
	Sham (*n* = 21)				
Jiang et al. (2022)	26 healthy adults	2 mA, 20 min 4x1 montage with center at primary motor cortex, M1 (C3); 3×1 montage with center at left DLPFC (F3)	3 sessions	Anodal	Two participants reported dizziness;
			2 active (M1 or DLPFC)		One participant reported very mild discomfort after all stimulus conditions
			1 sham		
Kold and Graven-Nielsen (2022)	40 healthy adults	2 mA, 20 min Montage of 7 electrodes targeting both left DLPFC and M1 (F3/C3)	3 sessions	Anodal	No adverse effects of the interventions were reported by participants
	Active (*n* = 20)				
	Sham (*n* = 20)				
Panico et al. (2022)	45 healthy adults	2 mA, 21 min 10 electrode montage with anodal or cathodal current distributed equally to the left PPC (P3) and right cerebellum (Cb)	1 session	Both	Weak itching sensation reported by all participants
	Anodal (*n* = 15)				
	Cathodal (*n* = 15)				
	Sham (*n* = 15)				
Stefano et al. (2022)	11 healthy adults	3 blocks of 2 min at each current intensity (1, 2, 3 mA) and an inter-stimulus interval of 5 min between blocks 3×1 montage centered at the right TPJ	3 sessions	Both	Stimulation was well tolerated across conditions and participants reported no significant discomfort related to the stimulation
			Anodal		
			Cathodal		
			Sham		
Xiong et al. (2023)	66 healthy females	2 mA, 20 min 4x1 montage with the central electrode targeting the anterior cingulate cortex (Fz)	1 session	Both	Itching and tingling (*n* = 50, 76%) were the most common side effects; other effects included skin redness, drowsiness, headache, dizziness, difficulty concentrating, and nausea
	Anodal (*n* = 22)				
	Cathodal (*n* = 23)				
	Sham (*n* = 21)				
Xiao et al. (2023)	16 young adults	2 mA, 20 min 4x1 montage with the anode centered over the sensorimotor cortex (Cz)	2 sessions	Anodal	No significant difference between groups in ratings of pain, burning or lightheadedness; HD-tDCS stimulation resulted in higher ratings or unpleasant tingling and itchiness compared to the sham group
			Active		
			Sham		
Berglund-Barraza et al. (2023)	28 young adults	1 mA, 20 min 4x1 montage with the anode centered over the preSMA (Fz)	1 session	Anodal	No significant difference between groups in ratings of itching, neck/scalp pain, tingling, burning sensation, headache, skin redness, sleepiness, trouble concentrating, mood change
	Active (*n* = 14)				
	Sham (*n* = 14)				
Johari and Berger (2023)	21 healthy adults	2 mA, 25 min 4x1 montage with the cathode centered over the right DLPFC (FC2)	2 sessions	Cathodal	Ratings of pain/unpleasantness were mild and did not differ significantly between groups at the beginning, halfway through, or at the end of the stimulation
			Cathodal		
			Sham		
Schroeder et al. (2024)	68 participants	1 mA, 30 min 4x1 montage with the cathode centered over the left DLPFC (F3) or left IFG (FC5)	1 session	Cathodal	Ratings of itching/tingling were higher in stimulation groups;
	Left IFG (*n* = 25)				Ratings of pain, burning, warmth/heat, iron taste, headache and exhaustion were mild and groups did not differ from each other
	Left DLPFC (*n* = 22)				
	Sham (*n* = 21)				
Shen et al. (2024)	16 healthy adults	2 mA, 20 min 4x1 montage with the anode centered at Cz	2 sessions	Anodal	Participants completed both conditions without reporting any serious adverse effects
			Active		
			Sham		
Imperio and Chua (2024)	36 participants	2 mA, 15 min 4x1 montage with the anode centered over the left (F3) or right (F4) DLPFC	3 sessions	Anodal	Participants indicated higher frequency of burning sensations in left compared to right DLPFC and sham. For all other sensations there were no significant differences between stimulation conditions
			Left DLPFC		
			Right DLPFC		
			Sham		
Tabari et al. (2024)	24 participants	2 mA, 25 min 4x1 montage with the central electrode targeting the left SMA (Cz)	3 sessions	Both	Most frequently reported sensations (pain/unpleasantness) in descending order of frequency were burning, stinging, and pins and needles; no significant differences found between the 3 stimulation conditions
			Anodal		
			Cathodal		
			Sham		
Otstavnov et al. (2024)	48 healthy participants	1 mA, 15 min 4x1 montage with the anode targeting the left DLPFC (F3) and the left PPC (P3)	1 session	Anodal	No differences between the three groups for all potential side effects (itching, pain, burning, heating, pinching, taste of iron, and fatigue)
	Left DLPFC/PPC (*n* = 17)				
	Left DLPFC (*n* = 16)				
	Sham (*n* = 15)				
Cacciamani et al. (2024)	62 participants	2 mA, 20 min 4x1 montage with the anode centered at the left LOC (PO7)	1 session	Anodal	Most common symptoms were scalp tingling, scalp warmth, and mild headache
	Active (*n* = 32)				
	Sham (*n* = 30)				
Kipping et al. (2024)	52 participants	2 mA, 20 min 4x1 montage targeting the right anterior insula	2 sessions	Anodal	Sensations experienced included tingling and itching Frequency and intensity of sensations were higher for active compared to sham group
			Active		
			Sham		
Wang L. et al. (2024)	25 participants	2 mA, 20 min 4x1 montage targeting the left DLPFC (F3)	3 sessions	Both	No adverse events were reported during stimulation, and no poststimulation side effects were reported
			Anodal		
			Cathodal		
			Sham		
Schmidt et al. (2024)	106 older adults	1 mA, 20 min 4x1 montage with the central electrode located at the right or left inferior frontal lobe (FC6/FC5) or right superior parietal lobe (P4)	1 session	Both	Few and mild side effects reported; occurrence of was similar in all groups; Ratings of side effects included headache, tingling, itching, burning, sleepiness, loss of focus, neck//scalp pain
	Cathodal				
	L. inferior frontal lobe (*n* = 15)				
	*R. inferior* frontal lobe (*n* = 16)				
	R. superior parietal lobe (*n* = 15)				
	Anodal				
	L. inferior frontal lobe (*n* = 15)				
	*R. inferior* frontal lobe (*n* = 15)				
	R. superior parietal lobe (*n* = 15)				
	Sham (*n* = 15)				
Fresnoza et al. (2024)	Exp. 1: 20 healthy young adults	1.5 mA, 10 min 4x1 montage targeting the left IFG (F7) in Exp. 1 and the left DLPFC (F3) in Exp. 2	3 sessions	Both	Reports of itching/tingling; no other adverse effects
	Exp. 2: 21 healthy young adults		Anodal		
			Cathodal		
			Sham		
Jin et al. (2025)	20 healthy participants	1 mA, 20 min 4x1 montage with the anode targeting the right primary motor cortex (C4)	3 sessions	Anodal	Tingling, itching, pain
			Concurrent tDCS		
			Prior-tDCS		
			Sham		

#### Adverse events by stimulation parameters

The intensity and duration of stimulation are two primary parameters that influence the incidence and nature of adverse events in HD-tDCS studies. Across the reviewed literature, stimulation currents ranged from 1 mA to 3 mA, with durations spanning 10 to 30 min. A consistent trend emerged in which higher intensities and longer durations were associated with a modest increase in the frequency of common adverse events, primarily tingling, itching, warmth, and mild headache. Yet even at higher intensities and longer durations adverse events were mainly mild in severity and transient.

Across studies administering HD-tDCS at intensities between 1 and 1.5 mA for durations of 10 to 20 min, the most common sensations were mild and transient including burning, tingling, and itching under the site of the electrodes (Caparelli-Daquer et al., 2012; Roy et al., 2014; Turski et al., 2017; Lenoir et al., 2017; Ke et al., 2019; Fresnoza et al., 2024; Jin et al., 2025). Occasional reports of headache were also noted, typically resolving without intervention (Turski et al., 2017). In most cases, adverse event ratings were described as mild (Huang et al., 2021; Schmidt et al., 2024; Schroeder et al., 2024), and comparisons between active and sham stimulation groups showed no significant differences across symptoms such as headache, neck/scalp pain, tingling, itching, burning, sleepiness, concentration, mood changes, and skin irritation (Gbadeyan et al., 2016b; Gbadeyan et al., 2016a; Pixa et al., 2017a; Martin et al., 2017a; Martin et al., 2017b; Perceval et al., 2017; Gbadeyan et al., 2019; Behroozmand et al., 2020; Splittgerber et al., 2020; Martin et al., 2021; Berglund-Barraza et al., 2023; Otstavnov et al., 2024; Hill et al., 2018; Naka et al., 2018). This finding was similarly observed at 1.5 mA (Hill et al., 2018; Naka et al., 2018; Hill et al., 2019; Guo et al., 2018). When participants did withdraw from a study, the primary reason cited was discomfort during stimulation (Hill et al., 2018).

In studies that did report differences between stimulation and sham groups at 1 mA, one study found that effects were limited to the first 5 min of stimulation, with ratings comparable to that of the sham group by the end of the stimulation session (Hill et al., 2017). Other studies found slightly higher ratings for specific sensations, such as scalp pain (Martin et al., 2017a; Martin et al., 2017b) or itching/tingling (Schroeder et al., 2024), in the active group, but overall adverse event profiles remained mild and comparable between conditions. Similar patterns were observed at 1.5 mA, with higher ratings of pain/discomfort (Sedgmond et al., 2020) and skin tingling (Lu et al., 2021) in the active compared to sham group, but no severe or lasting effects.

In some cases the sham group has been found to report higher sensation ratings compared to the active group. For example, one study found that participants in the sham group reported increased ratings of the experience of tingling compared to that of the active group, although ratings of a burning sensation were higher for the active group compared to the sham group (To et al., 2018). In other cases, no evidence for the effect of stimulation on adverse events has been found at all for 1 mA (Martin et al., 2019b; Martin et al., 2019a; Stefano et al., 2022), 1.5 mA (Wen et al., 2019; Dai et al., 2021), 2 mA (Stefano et al., 2022; Flood et al., 2016; Shen et al., 2016; Kumar et al., 2020; Xiao et al., 2020; Wan et al., 2021; Kold & Graven-Nielsen, 2022; Kold & Graven-Nielsen, 2021; Shen et al., 2024; Wang L. et al., 2024) or 3 mA (Stefano et al., 2022).

At 2 mA, HD-tDCS was again associated with sensations such as tingling, burning, and itching (Kuo et al., 2013; Nikolin et al., 2019; Borckardt et al., 2012; Zhou et al., 2019; Xiong et al., 2023; Tabari et al., 2024; Kipping et al., 2024). While most reports were cited as being “weak” (Panico et al., 2022) or “mild” (Choi and Perrachione, 2019; Gan et al., 2019; Ballard et al., 2021; Jiang et al., 2022; Johari and Berger, 2023; Cacciamani et al., 2024), in some cases they were reported as “moderate” (Chua et al., 2017; Choi and Perrachione, 2019; Gan et al., 2019; Weintraub-Brevda and Chua, 2019) or “severe” (Chua et al., 2017; Gan et al., 2019; Weintraub-Brevda and Chua, 2019; Donaldson et al., 2019). Across most trials, comparisons between active and sham conditions revealed no significant differences in reported symptoms (da Silva Machado et al., 2021; Behroozmand et al., 2020; Tabari et al., 2024; Choi and Perrachione, 2019; Johari and Berger, 2023; Weintraub-Brevda and Chua, 2019; Garnett and den Ouden, 2015; Price et al., 2016; Besson et al., 2019; Lang et al., 2019; Hartmann et al., 2020), and ratings of discomfort, when present, tended to be low. In one study in which participants were administered stimulation at 0.5 mA and 2 mA, perceived sensation ratings were found to be higher in the 2 mA vs. 0.5 mA condition at 30 s after stimulation start, but converged over time (Brunyé et al., 2014). Some studies observed slightly elevated ratings of tingling/itchiness (Xiao et al., 2023), pain (Nikolin et al., 2019; Savic et al., 2019) or sleepiness (Reckow et al., 2018) ratings in the active condition, while others observed that the frequency (Kipping et al., 2024) or strength (Kipping et al., 2024; Lerner et al., 2021) of adverse effects were significantly higher for the stimulation groups compared to the sham group. While a couple of self-resolving headaches were reported (Nikolin et al., 2015), participants generally tolerated stimulation at 2 mA well. When participants withdrew at this intensity, it was usually due to sensations described as “needle-prickling” (Nikolin et al., 2015), unpleasant (Chua et al., 2017), stinging/burning (Nikolin et al., 2019), or general discomfort (Ballard et al., 2021).

While severe adverse events were infrequently reported, they were not entirely absent. Severe sensations, such as tingling, burning, headache, sleepiness and redness, were observed in a small number of participants and were typically associated with 2 mA stimulation using a 4 × 1 ring montage for 20 min at frontal, temporal or parietal sites (Chua et al., 2017; Gan et al., 2019; Weintraub-Brevda and Chua, 2019; Donaldson et al., 2019). For example, one study reported multiple instances of severe burning, headache, tingling, and redness across 39 participants, with one participant withdrawing prior to stimulation due to discomfort (Chua et al., 2017). Another study noted six reports of severe adverse events among 60 participants, including two in the sham group (Weintraub-Brevda and Chua, 2019). While severe adverse events were reported in these two studies, it is unclear how many of these reports came from the same participant. In studies where reporting is clearer, severe side effects were limited to one or two participants. For example, one study reported that one out of 22 participants experienced severe tingling (Gan et al., 2019) while another reported two of 53 participants temporarily ceased stimulation due to discomfort but resumed after a short 1–3 min break (Donaldson et al., 2019). In most cases, even participants experiencing stronger sensations completed the study, and reported symptoms resolved quickly.

Fewer studies have explored stimulation above 2 mA, but preliminary findings indicate that tolerability remains high. For example, in studies administering 3 or 4 mA over 20–30 min, no serious adverse events were observed (Reckow et al., 2018). In one large-scale study involving over 3,000 HD-tDCS sessions in older adults, the vast majority of sensations were rated as “none” or “mild,” regardless of whether the stimulation was active or sham (El Jamal et al., 2023). Interestingly, some mild sensations, such as tingling or itching, were reported more frequently at lower intensities (< 2 mA), while skin redness was slightly more common at higher intensities (> 3 mA). These findings suggest that HD-tDCS is well tolerated even at intensities up to 4 mA, although further research is needed to confirm these outcomes across broader populations.

#### Adverse events by number of HD-tDCS sessions

Most HD-tDCS studies in healthy individuals have employed single-session protocols and consistently report mild, short-lived sensations such as tingling, itching, burning and prickling (Lenoir et al., 2017; Huang et al., 2021; To et al., 2018; Wen et al., 2019),—if any—without adverse effects on participant well-being or task engagement. In many of these studies, no significant differences in adverse event ratings are found between active and sham stimulation groups (Schmidt et al., 2024; Behroozmand et al., 2020; Berglund-Barraza et al., 2023; Otstavnov et al., 2024; Naka et al., 2018; Guo et al., 2018; Choi and Perrachione, 2019; Weintraub-Brevda and Chua, 2019; Lang et al., 2019; Savic et al., 2019; Pixa et al., 2017b). When differences do arise, they typically involve slightly higher ratings of pain (Nikolin et al., 2019) or discomfort (Lerner et al., 2021) in the active group, while overall sensation ratings remain mild. For example, one study reported increased itching and tingling during active stimulation but found no differences in ratings of other sensations such as pain, burning, warmth/heat, iron taste, exhaustion and headache (Schroeder et al., 2024).

Multi-session protocols in which participants experience both active and sham stimulation provide further insight into the tolerability of HD-tDCS. These studies frequently report no significant differences in the incidence (da Silva Machado et al., 2021; Splittgerber et al., 2020; Martin et al., 2021) or intensity (Gbadeyan et al., 2016b; Gbadeyan et al., 2016a; Gbadeyan et al., 2019; Tabari et al., 2024; Garnett and den Ouden, 2015; Price et al., 2016; Hartmann et al., 2020) of adverse event ratings across conditions, including sensations such as pain, tingling, itching, burning, sleepiness, concentration, mood changes and skin irritation. Other studies have found no difference in cutaneous sensations in general (Hill et al., 2018; Hill et al., 2019; Besson et al., 2019). In cases where adverse events are assessed over the course of a stimulation session, one study observed an initial difference in ratings of itchiness between groups that was found to resolve over the course of the 15-min stimulation session (Hill et al., 2017). Another study measured ratings of pain and unpleasantness at the beginning, halfway through, or at the end of the stimulation and found that ratings did not differ between groups across time (Johari and Berger, 2023). While some studies do show higher ratings in the active group compared to the sham group for adverse events such as scalp pain (Martin et al., 2017a; Martin et al., 2017b), pain/discomfort (Sedgmond et al., 2020), unpleasant tingling/itchiness (Kipping et al., 2024; Xiao et al., 2023), and for burning (Imperio and Chua, 2024), a number of studies also report no adverse events at all (Martin et al., 2019b; Martin et al., 2019a; Stefano et al., 2022; Dai et al., 2021; Flood et al., 2016; Shen et al., 2016; Xiao et al., 2020; Wan et al., 2021; Shen et al., 2024; Wang L. et al., 2024), underscoring the favorable safety profile of HD-tDCS.

Repeated-session protocols, where participants receive the same stimulation condition over several sessions, also support the tolerability of HD-tDCS. Across studies ranging from 3 to 20 sessions and intensities between 1 to 2 mA, reported adverse events are typically mild, including sensations such as transient tingling, skin redness, slight discomfort (Lu et al., 2021; Ke et al., 2019), or none at all (Kold and Graven-Nielsen, 2021; Kold and Graven-Nielsen, 2022). In one study involving 20 sessions, no participants withdrew, and no lasting effects were observed (Turski et al., 2017). Another large study involving varied session counts and stimulation intensities in older adults reported no serious adverse events across more than 100 participants (Reckow et al., 2018). While isolated reports of severe sensations (e.g., burning or headache) do exist (Chua et al., 2017), including some in sham groups, such instances are uncommon and typically do not result in withdrawal from the study. Overall, these findings suggest that HD-tDCS is well tolerated in healthy individuals, even with repeated exposure, although continued large-scale studies with systematic tracking of adverse effects are warranted.

#### Adverse events by polarity: anodal vs. cathodal

A few studies using either a 3×1 or 4×1 ring montage have employed both anodal and cathodal HD-tDCS to investigate polarity-specific effects. Across these studies, the most commonly reported sensations were itching and tingling under the electrodes (Kuo et al., 2013; Caparelli-Daquer et al., 2012; Roy et al., 2014; Fresnoza et al., 2024). In studies that directly compared adverse effects across anodal, cathodal, and sham conditions, no significant differences were observed in participants’ ratings of pain or unpleasantness (Tabari et al., 2024), indicating similar tolerability across polarities. Other studies simply noted that participants tolerated both forms of stimulation well, with no adverse events reported (Stefano et al., 2022; Shen et al., 2016; Wang L. et al., 2024). Collectively, these findings suggest that stimulation polarity does not substantially affect tolerability, although further research is warranted to confirm this conclusion.

Overall, the consistency of these findings across different stimulation parameters and populations of healthy adults—including older adults—supports the tolerability and safety of HD-tDCS in non-clinical research settings. However, few studies have systematically investigated the tolerability of HD-tDCS, which has prevented the widespread application of it to the intervention of neurologic or psychiatric disorders.

### Clinical populations

HD-tDCS has been used in a wide range of clinical populations including patients with fibromyalgia (Villamar et al., 2013a; Villamar et al., 2013b; Castillo-Saavedra et al., 2016), aphasia (Richardson et al., 2015; Fiori et al., 2019; Shah-Basak et al., 2020), chronic myofascial temporomandibular disorder pain (Donnell et al., 2015), tinnitus (Jacquemin et al., 2021; Jacquemin et al., 2018; Shekhawat et al., 2016; Shekhawat and Vanneste, 2018; Henin et al., 2016; Cardon et al., 2022), epilepsy (Karvigh et al., 2017; Ng et al., 2023; Roshan et al., 2024), schizophrenia (Sreeraj et al., 2018; Xu et al., 2023; Yeh et al., 2025), stroke (Bao et al., 2019; Hu et al., 2024), depression (Wong et al., 2019), traumatic brain injury (Motes et al., 2020; Chiang et al., 2021), chronic low back pain (McPhee and Graven-Nielsen, 2021), diabetes mellitus (Filipas et al., 2022), obsessive compulsive disorder (Wang Y. et al., 2024), Alzheimer’s disease (Lobue et al., 2025) and methamphetamine use disorder (Hou et al., 2025). Across these diverse samples, HD-tDCS has been shown to be generally well tolerated, with reported adverse events typically limited to transient sensations such as tingling, itching, or mild burning (Table 2). Importantly, no serious adverse effects have been reported in any of the reviewed studies, underscoring the safety profile of HD-tDCS in clinical contexts.

**Table 2:** Study design and adverse event reports for clinical populations.

	Sample size and population	Stimulation parameters	Number of sessions	Polarity	Reported adverse events
Villamar et al. (2013a, 2013b)	18 fibromyalgia patients	2 mA, 20 min 4x1 montage with center at left M1	3 sessions	Both	Mild-to-moderate tingling or itching during both active and sham stimulation; sensations faded within minutes
			Anodal		
			Cathodal		
			Sham		
Richardson et al. (2015)	8 patients with chronic stroke-induced aphasia	1 mA, 20 min individualized targeting	11 sessions	Both	Tingling, burning
Donnell et al. (2015)	24 females with chronic myofascial temporomandibular disorder pain	2 mA, 20 min modified 2×2 HD-tDCS montage with electrode centered over caudal portion of putative M1	5 sessions	Both	Low ratings of headache, neck / scalp pain, scalp burning, tingling, skin redness, sleepiness, trouble concentrating and mood change for both active and sham groups; low rate of adverse events; Mild in nature
	Active (*n* = 12)				
	Sham (*n* = 12)				
Castillo-Saavedra et al. (2016)	14 fibromyalgia patients	2 mA, 20 min 4x1 montage with anode centered at the left primary motor cortex, M1 (C3)	10 sessions initially;	Anodal	Tingling (73.33%)
			Up to 26 sessions total		Mild headache (40%)
					Mild pain in stimulation area (40%)
					Skin redness in stimulation area (26.66%)
Shekhawat et al. (2016)	27 participants with chronic tinnitus	1 vs. 2 mA, 10 vs. 20 min 4x1 montage with anode centered at the left temporoparietal area, LTA (between C3/P5) or right DLPFC (F4)	2 sessions	Anodal	Reported sensations of headache, neck/scalp pain, scalp burn, tingling sleepiness, trouble concentrating and mood change; All brief and at onset of stimulation; Experienced mainly in the 2 mA/10 min condition
			LTA Active		
	LTA/DLPFC (*n* = 13)		DLPFC Active		
	DLPFC/LTA (*n* = 14)				
Henin et al. (2016)	14 patients with chronic tinnitus	2 mA, 20 min 2×2 stimulation delivered bilaterally over prefrontal lateral cortex and auditory cortex with opposing polarities	2 sessions	Both	No side effects reported by participants
Karvigh et al. (2017)	10 adult patients with intractable lateral frontal lobe epilepsy	2 mA, 20 min 4x1 montage with cathode centered over the epileptogenic zone	10 sessions	Cathodal	One report of a mild headache; All patients tolerated stimulation without any adverse reaction
Shekhawat et al. (2016)	13 patients with continuous chronic tinnitus	2 mA, 20 min 4x1 montage with anode centered at the right DLPFC (F4)	2 sessions	Anodal	No difference between active/sham groups in ratings of headache, neck/scalp pain, scalp burns, tingling, sleepiness, trouble concentrating, or acute mood change; Any experienced effects were transient and did not persist following stimulation
			Active		
			Sham		
Jacquemin et al. (2018)	39 patients with chronic nonpulsatile tinnitus	2 mA, 20 min 4x1 montage with anode centered at the right DLPFC (F4)	8 sessions	Anodal	Fewer sensations reported for HD-tDCS compared to tDCS treatment; stimulation well tolerated by all participants
Sreeraj et al. (2018)	19 patients with schizophrenia	-2 MA, 20 min 4x1 ring montage with cathode centered at the left temporoparietal junction (CP5)	10 sessions	Cathodal	1 patient reported headache, scalp pain, tingling, redness, sleepiness; 3 patients reported burning sensation; 4 patients reported skin lesions (healed by next day); 7 patients reported itching
Bao et al. (2019)	11 patients with chronic stroke	1 mA, 10 min 4x1 montage with electrode centered at ipsilesional motor cortex (C3/C4)	3 sessions	Both	Itching/tingling sensation on the scalp under the central electrode reported especially in the ramping up and down stimulation periods
			Anode		
			Cathode		
			Sham		
Fiori et al. (2019)	20 patients with chronic aphasia	1 or 2 mA, 20 min 4x1 ring montage with cathode on right homolog of Broca’s area (F4)	10 sessions	Cathodal	No difference in ratings of itchiness, pain, burning, warmth/heat, pinching, or fatigue between stimulation groups
	1 mA group (*n* = 10)		Active (5 sessions)		
	2 mA group (*n* = 10)		Sham (5 sessions)		
Wong et al. (2019)	15 participants with late-life depression	2 mA, 20 min 4x1 montage with anode centered at the left DLPFC (F3)	10 sessions	Anodal	9 participants reported mild side effects, such as tingling, itchiness, and mild skin redness at the stimulation site
Motes et al. (2020)	14 veterans with TBI	1 mA, 20 min 4x1 montage with anode targeting the pre-supplementary motor area/dorsal anterior cingulate cortex (preSMA/dACC; FCZ)	10 sessions	Anodal	Stimulation was tolerated well
	Active (*n* = 8)				
	Sham (*n* = 6)				
Shah-Basak et al. (2020)	11 patients with aphasia	2 mA, 20 min 3×1 montage with either left anodal or right cathodal stimulation dependent on patients’ brain abnormalities	3 sessions	Both	2 patients reported minor itchiness and neck pain/stiffness during anodal- and cathodal-tDCS; 1 patient reported neck pain/stiffness during sham tDCS
			Left Anodal		
			Right Cathodal		
			Sham		
McPhee and Graven-Nielsen (2021)	12 Chronic Low Back Pain Patients	2 mA, 20 min 4x1 ring configuration with anode centered at the medial prefrontal regions (Fz)	6 sessions	Anodal	No differences between groups in sensations reported during stimulation (heat/warmth/burning, itching, tingling, pins and needles/prickling) or side effects reported poststimulation; 1 participant ceased the active stimulation at 10 min on day 3 due to intolerable scalp sensation
			Active (3 sessions)		
			Sham (3 sessions)		
Chiang et al. (2021)	15 US veterans with chronic TBI	1 mA, 20 min 4x1 ring configuration with anode centered at pre-SMA/dACC (Fz)	10 sessions	Anodal	No differences in pain/discomfort were found when levels were evaluated before and after each session
	Active (*n* = 18)				
	Sham (*n* = 7)				
Jacquemin et al. (2021)	117 patients with chronic tinnitus	2 mA, 20 min 4x1 montage with center at right DLPFC (F4)	6 sessions	Anodal	5 patients reported one-time effects such as tingling, itchiness, headache, burning, feeling blurry; 2 patients reported headaches a few hours after each session; 1 patient reported aggravated tinnitus symptoms; Side-effects were tolerable
Filipas et al. (2022)	11 individuals diagnosed with type 1 diabetes mellitus	1.5 mA, 20 min 8 electrodes in two 3×1 arrays with the two anodes over the DLPFC (F3 and F4)	2 sessions	Anodal	No side effects reported by participants during or after sessions
			Active		
			Sham		
Cardon et al. (2022)	77 patients with chronic subjective tinnitus	2 mA, 30 min 10 electrodes in two 4x1 arrays with the two anodes over the rDLPFC (F4) and left temporal area (CP5)	6 sessions	Anodal	8 participants (10.4%) reported side effects. Active: 2 reported light, transient headaches post-stimulation; Sham: 4 reported light transient headaches, 1 more serious migraine-lie headache, 1 reported tingling sensations in the extremities following stimulation; No difference between groups in the presence of side effects
	38 Active				
	39 Sham				
Xu et al. (2023)	56 patients with chronic schizophrenia	2 mA, 20 min 4x1 montage with center at left DLPFC (F3)	1 session	Anodal	No differences between active and sham groups in either the strength or the cognitive impact of adverse sensations
	Active (*n* = 28)				
	Sham (*n* = 28)				
Ng et al. (2023)	10 patients diagnosed with refractory status epilepticus	2 mA, 20 min individualized targeting	1–10 sessions (varied by participant)	Cathodal	No reported adverse events over 32 stimulation sessions in 10 patients
Roshan et al. (2024)	12 adults diagnosed with drug-resistant left lateral frontal lobe epilepsy	2 mA, 20 min 4x1 montage with cathode on C3	10 sessions	Cathodal	Mild skin redness and itching in the stimulated area; no significant adverse effects reported during or after stimulation sessions until the one-month follow-up
Hu et al. (2024)	18 chronic stroke survivors	Up to 4 mA, 20 min individualized targeting	20 sessions	Both	Reported minor side effects occurred with similar incidence in both groups (*p* = 0.637)
	Active (*n* = 8)				
	Sham (*n* = 10)				
Wang Y. et al. (2024)	44 patients with obsessive compulsive disorder	1.5 mA, 20 min 4x1 montage with the cathode targeting the right OFC (FP2)	10 sessions	Cathodal	No significant differences between groups were found in ratings of headache, neck pain, scalp tingling, stinging sensation, itching, burning sensation, skin redness, drowsiness, excessive worrying, intense mood change
	Active (*n* = 23)				
	Sham (*n* = 21)				
Yeh et al. (2025)	59 patients with schizophrenia	2 mA, 20 min 4x1 montage with the anode targeting the left DLPFC (F3)	10 sessions	Anodal	Side effects included tingling, sleepiness, headache, skin redness, burning sensation, dizziness and itching; Ratings of tingling were higher in the active compared to the sham group
	Active (*n* = 30)				
	Sham (*n* = 29)				
Lobue et al. (2025)	25 patients diagnosed with Alzheimer’s disease	1 or 2 mA, 20 min 4x1 montage with the anode targeting the medial prefrontal cortex (Fz)	10 sessions	Anodal	Tingling (88–100%), itching (44–60%), and burning sensations (33–55%) on the scalp were commonly reported and rated as mild to moderate
	1 mA group (*n* = 10)				
	2 mA group (*n* = 10)				
	Sham group (*n* = 5)				
Hou et al. (2025)	60 patients with methamphetamine use disorder	1.5 mA, 20 min 4x1 montage with the anode targeting the left DLPFC (F3) or right DLPFC (F4)	8 sessions	Anodal	Slight redness and itchiness of the scalp following stimulation that disappeared within 15 min
	Left DLPFC Active (*n* = 20)				
	Right DLPFC Active (*n* = 20)				
	Sham (*n* = 20)				

#### Adverse events by stimulation parameters

In clinical populations, HD-tDCS has most commonly been administered at 2 mA (Villamar et al., 2013b; Castillo-Saavedra et al., 2016; Wong et al., 2019; Jacquemin et al., 2021; Jacquemin et al., 2018; Shah-Basak et al., 2020; Donnell et al., 2015; Shekhawat and Vanneste, 2018; Henin et al., 2016; Cardon et al., 2022; Karvigh et al., 2017; Ng et al., 2023; Roshan et al., 2024; Sreeraj et al., 2018; Xu et al., 2023; Yeh et al., 2025; McPhee and Graven-Nielsen, 2021), though some studies have used lower intensities of 1 mA (Richardson et al., 2015; Bao et al., 2019; Motes et al., 2020; Chiang et al., 2021) or 1.5 mA (Filipas et al., 2022; Wang Y. et al., 2024; Hou et al., 2025). Across all intensities, the most frequently reported sensations include tingling and itching, with occasional reports of burning (Jacquemin et al., 2021; Richardson et al., 2015; Sreeraj et al., 2018; Yeh et al., 2025; Lobue et al., 2025) and headache (Castillo-Saavedra et al., 2016; Jacquemin et al., 2021; Cardon et al., 2022; Karvigh et al., 2017; Sreeraj et al., 2018; Yeh et al., 2025). These effects are typically mild and transient. Several studies report no adverse effects at 1 mA (Motes et al., 2020; Chiang et al., 2021), 1.5 mA (Filipas et al., 2022), or even at 2 mA (Henin et al., 2016; Ng et al., 2023). In direct comparisons between intensities, one study involving patients with tinnitus found that tingling, sleepiness, and mild scalp discomfort were more frequently reported at 2 mA than 1 mA, though all reported sensations across conditions were brief and occurred primarily at the onset of stimulation (Shekhawat et al., 2016). Another study in patients with aphasia reported no significant differences in adverse effects between 1 and 2 mA stimulation (Fiori et al., 2019), and similar results were found in patients with Alzheimer’s disease, where mild to moderate sensations were observed across both intensities (Lobue et al., 2025).

While the majority of studies describe adverse effects as “mild” (Villamar et al., 2013a; Castillo-Saavedra et al., 2016; Wong et al., 2019; Shah-Basak et al., 2020; Karvigh et al., 2017; Roshan et al., 2024; Hu et al., 2024; Lobue et al., 2025), a few have reported moderate discomfort (Villamar et al., 2013a; Lobue et al., 2025) and in rare instances, more severe symptoms have been noted. For example, in one study applying 2 mA stimulation for 30 min to the right DLPFC and left temporal area using two 4 × 1 arrays (10 electrodes total), a single participant out of 77 with chronic subjective tinnitus experiences a more serious migraine-like headache (Cardon et al., 2022). This event occurred under a longer than typical 30 min protocol with multiple stimulation sites, suggesting that extended duration and multi-target montages may modestly increase the likelihood of adverse events, though additional research is needed to confirm this. Studies using intensities above 2 mA are limited, but one investigation using 4 mA in chronic stroke survivors over 20 sessions reported only minor side effects, with no significant difference in adverse event rates between active and sham conditions (Hu et al., 2024). These findings suggest that, with appropriate monitoring and protocol design, HD-tDCS may be safely administered at higher intensities in therapeutic contexts.

A critical consideration in clinical populations is whether HD-tDCS exacerbates existing symptoms. Encouragingly, the technique has generally been found to either improve clinical outcomes or have negligible negative impact. Reports of symptom aggravation are exceedingly rare. One study involving 117 patients with chronic tinnitus noted a single case in which symptoms were worsened (Jacquemin et al., 2021). Although uncommon, such findings underscore the importance of routine monitoring, particularly in multi-session treatment protocols, to ensure patient safety and responsiveness to the intervention.

#### Adverse events by number of HD-tDCS sessions

Unlike studies in healthy individuals, HD-tDCS research in clinical populations more commonly involves multi-session protocols. Although the number of sessions varies widely, from a single exposure to 20 or more sessions, repeated stimulation generally does not appear to increase the occurrence or severity of adverse events. Across studies administering 10 or more sessions, participants most often report only mild and transient sensations, such as tingling or itching (Castillo-Saavedra et al., 2016; Wong et al., 2019; Richardson et al., 2015; Karvigh et al., 2017; Roshan et al., 2024; Sreeraj et al., 2018; Hu et al., 2024; Lobue et al., 2025). For example, one study involving individuals with chronic traumatic brain injury found no significant change in self-reported pain or discomfort across 10 sessions, suggesting stable tolerability over time (Chiang et al., 2021). While rare, instances of early discontinuation due to discomfort have been documents. In one study, a participant with chronic low back pain discontinued stimulation on day three due to scalp discomfort, despite tolerating earlier sessions (McPhee and Graven-Nielsen, 2021). These findings suggest that HD-tDCS is well tolerated across repeated sessions in clinical populations, though individual variability in sensitivity should be monitored.

#### Adverse events by polarity: anodal vs. cathodal

Relatively few studies in clinical populations have directly compared the side effects associated with anodal versus cathodal HD-tDCS. However, existing evidence in studies employing both 3×1 and 4×1 ring montages suggests that polarity has minimal influence on the frequency or severity of adverse events. In patients with fibromyalgia, both anodal and cathodal stimulation produced similar mild-to-moderate sensations, such as tingling and itching (Villamar et al., 2013a). Comparable findings were reported in individuals with chronic stroke, where participants experienced transient scalp sensations, primarily tingling and itching under the central electrode, at the beginning and end of both stimulation conditions (Bao et al., 2019). Similarly, a study involving patients with aphasia noted only minor discomfort during both anodal and cathodal protocols (Shah-Basak et al., 2020). Although further research is needed to confirm these patterns across a broader range of clinical conditions, current findings indicate that HD-tDCS polarity does not substantially affect tolerability in clinical populations.

## Exploratory analysis of AE frequency with stimulation parameters

Although most studies report only mild adverse events, it is equally important to consider research in which participants experience comparable stimulation parameters without any reported side effects. In both healthy and clinical populations, studies using 2 mA for 20 min consistently report no side effects (Shen et al., 2016; Xiao et al., 2020; Wan et al., 2021; Kold and Graven-Nielsen, 2021; Kold and Graven-Nielsen, 2022; Wang L. et al., 2024; Ng et al., 2023). In several of these studies participants experienced both active and sham conditions and still reported no adverse effects, effectively serving as their own controls and underscoring the robust tolerability of HD-tDCS (Martin et al., 2019a; Stefano et al., 2022; Flood et al., 2016; Shen et al., 2016; Xiao et al., 2020; Wan et al., 2021; Shen et al., 2024; Henin et al., 2016; Filipas et al., 2022). Additionally, a study administering multiple intensities (1 mA, 2 mA, 3 mA) in short blocks using a 3×1 montage targeting the right TPJ reported no adverse events (Stefano et al., 2022). These findings indicate that although mild adverse events may be more likely at higher intensities or longer durations, their relationship is not conclusive.

To explore whether higher stimulation parameters were associated with increased adverse event (AE) reporting, a simple exploratory analysis was conducted using data extracted from the reviewed studies. Each study was coded for the presence or absence of any reported AE or any significant group differences, and stimulation parameters were classified by intensity (≤1.5 mA vs. ≥2 mA), duration (≤20 min vs. >20 min), and polarity (anodal, cathodal, or both).

Among 33 studies in clinical populations, AE frequency was not significantly different across studies that used different stimulation intensity, duration, nor polarity (*p* > 0.18). When studies were compared that used different stimulation intensity, 54.5% of those using <2 mA reported at least one mild AE, compared to 77.3% of studies using ≥2 mA. This difference was not statistically significant (χ^2^(1) = 1.793, *p* = 0.181). When duration was examined, 100% of studies using <20 min reported at least one mild AE compared to 66.7% of those using ≥20 min (χ^2^(1) = 1.435, *p* = 0.231). When polarity was considered, 73.7% of anodal studies reported at least one mild AE, compared to 42.9% of cathodal studies and 85.7% of those using both polarities (χ^2^(1) = 3.381, *p* = 0.184).

Likewise, no significant differences were observed when AE reporting was compared across 80 studies in healthy populations (*p* > 0.32). 55.6% of those using <2 mA reported at least one mild AE, compared to 56.8% of studies using ≥2 mA (χ^2^(1) = 0.013, *p* = 0.910). Similarly, 47.4% of studies using <20 min reported at least one mild AE compared to 59% of those using ≥20 min (χ^2^(1) = 0.799, *p* = 0.371). When polarity was examined, 54.2% of anodal studies reported at least one mild AE, compared to 33.3% of cathodal studies and 65.4% of those using both polarities (χ^2^(1) = 2.247, *p* = 0.325).

Taken together, these findings indicate that within the commonly used stimulation ranges (1–2 mA, 10–30 min), HD-tDCS is well tolerated in both clinical and healthy participants, and parameter variations do not appear to systematically alter the likelihood of AE reporting.

## Inconsistent adverse event reporting in the literature

Despite overall support for the tolerability of HD-tDCS, adverse event reporting remains inconsistent across both healthy and clinical populations. While some studies provide detailed accounts of stimulation related sensations, even if mild (Castillo-Saavedra et al., 2016; Jacquemin et al., 2021; Shah-Basak et al., 2020; Cardon et al., 2022; Sreeraj et al., 2018; Lobue et al., 2025), others merely state that the procedure was “well-tolerated” or that no adverse effects were reported (Ng et al., 2023; Motes et al., 2020; Filipas et al., 2022), without offering specifics or describing how side effects were assessed. A particularly concerning issue is that many studies describe stimulation parameters and experimental procedures in great detail but fail to mention adverse effects or participant tolerability altogether. In some cases it is stated that tolerability or symptom assessment was part of the methodology (Donaldson et al., 2018; Gallo et al., 2018; Chen et al., 2019; Gaynor and Chua, 2019; de Boer et al., 2020; Masina et al., 2021; Erker et al., 2024), such as by including an adverse event questionnaire or participant interviews, but these results are not reported in the findings or discussion.

In cases in which adverse events are reported, two main approaches are typically used. Some studies present adverse events on an individual basis, detailing the symptoms experienced by each participant and their perceived severity, while others summarize adverse events at the group level to compare active and sham stimulation conditions. Assessment methods also vary, from standardized questionnaires that quantify predefined adverse events to open-ended reports in which participants simply indicate whether they experienced any sensation. As a result of this heterogeneity, comparing the frequency and severity of adverse events across studies is challenging and is limited to general reports of sensations. This variability highlights the need for standardized adverse event assessment tools to enable more consistent comparisons across HD-tDCS research. Despite this variability, among the 39 studies reporting adverse events in Tables 1, 2, the most common sensations were tingling (85%), itching (69%), and burning (44%).

This lack of standardized adverse event reporting poses a significant challenge for comprehensive safety evaluation. The variability in language (e.g., “discomfort,” “pain,” “burning”) and the inconsistent timing of assessments (during stimulation, immediately post-session, or at follow-up) further complicates the interpretation and comparison of side effect profiles across clinical studies. Moreover, the use of different scales—or the absence of any scale—makes it difficult to gage the true incidence and impact of minor but clinically relevant sensations. These issues are especially concerning in the context of clinical populations, where HD-tDCS is used as a non-pharmacological alternative or adjunct to traditional treatments. Given that one of the main appeals of HD-tDCS in these groups lies in it being non-invasive and low-risk, systematic safety reporting is necessary to support its clinical adoption, regulatory approval, and patient confidence. The underreporting of adverse effects—even if mild or absent—can inadvertently undermine the very rationale for using HD-tDCS in these populations.

Additionally, the definitions for weak, mild, moderate and severe used in each study are typically based on participants’ subjective ratings, often collected with Likert-type or numeric rating scales, but the precise definitions are not always provided. This limits the extent to which direct comparisons can be made across studies. Despite this variability, the majority of reports converge in describing adverse events as mild and transient, with very few instances described as severe.

To advance the field, future studies should adopt standardized protocols for adverse event reporting. This includes predefining tolerability metrics including explicit definitions of severity levels, administering structured and validated side effect questionnaires—such as the tDCS adverse effects questionnaire proposed by Brunoni et al. (2011), and explicitly detailing both the presence and absence of side effects across all stimulation conditions. Comprehensive and systematic reporting will not only improve the interpretability and comparability of findings but will also help build a robust evidence base for HD-tDCS as a safe and effective tool for both research and clinical practice.

## Conclusion

Across both healthy and clinical populations, HD-tDCS has consistently demonstrated a favorable safety and tolerability profile. The vast majority of reported adverse events are mild, transient and comparable to those observed with conventional tDCS, typically limited to sensations such as tingling, itching, or slight burning at the electrode sites. Serious side effects are exceedingly rare, and isolated reports of severe adverse events, such as burning, tingling, or headache, tended to occur under conditions involving higher current intensities, longer durations, or multi-site montages, and symptoms typically resolved quickly without lasting effects. Across a wide range of stimulation parameters and clinical conditions, HD-tDCS has demonstrated to be a well-tolerated, non-invasive technique, supporting its use as a safe and reliable tool for both research and therapeutic applications.
